# Redefining pediatric leukemia care – innovations in risk assessment and targeted treatment: a narrative review

**DOI:** 10.1097/MS9.0000000000004742

**Published:** 2026-01-21

**Authors:** Emmanuel Ifeanyi Obeagu

**Affiliations:** Department of Molecular Medicine and Haematology, School of Pathology, Faculty of Health Sciences, University of the Witwatersrand, Johannesburg, South Africa

**Keywords:** acute lymphoblastic leukemia, acute myeloid leukemia, childhood leukemia, risk stratification, targeted therapy

## Abstract

Pediatric leukemia, primarily comprising acute lymphoblastic leukemia (ALL) and acute myeloid leukemia, represents approximately 30% of childhood cancers worldwide. Over the past decade, advances in molecular and genetic profiling, including next-generation sequencing and minimal residual disease monitoring, have refined risk stratification and enabled individualized treatment strategies. Integrating these approaches into clinical practice has improved survival rates, particularly for high-risk patients, by guiding therapy intensity and informing targeted interventions. Targeted therapies, such as tyrosine kinase inhibitors for Philadelphia chromosome-positive ALL, Chimeric Antigen Receptor T cell therapy for relapsed or refractory cases, and monoclonal antibodies like blinatumomab and inotuzumab ozogamicin, have transformed treatment outcomes while reducing chemotherapy-related toxicity. Despite these advances, challenges remain, including the development of therapy resistance (e.g., BCR-ABL1 and FLT3 mutations) and long-term adverse effects such as cardiotoxicity and secondary malignancies. This narrative review summarizes recent innovations in risk assessment and targeted therapies, highlights current challenges, and discusses future directions to optimize personalized pediatric leukemia care.

## Introduction

Pediatric leukemia, a hematologic malignancy, remains one of the most common cancers in children and adolescents worldwide, accounting for approximately 30% of childhood malignancies^[1,2]^. Among these, acute lymphoblastic leukemia (ALL) constitutes roughly 75% of cases, while acute myeloid leukemia (AML) accounts for most of the remaining diagnoses. Over the past several decades, significant improvements in chemotherapy, supportive care, and risk-adapted therapy have increased 5-year survival rates for ALL from approximately 60% in the 1970s to over 90% today. However, high-risk subtypes and relapsed disease continue to present considerable clinical challenges, with survival rates remaining substantially lower in these populations.^[3–6]^ Traditionally, risk stratification relied on clinical and laboratory parameters such as age at diagnosis, white blood cell (WBC) count, and cytogenetic features [e.g., t(12;21), t(9;22)][7]. While these factors provide valuable prognostic information, they are insufficient for fully capturing disease heterogeneity and predicting relapse, particularly in high-risk patients[8]. The advent of molecular and genomic profiling, including next-generation sequencing (NGS), fluorescence in situ hybridization (FISH), and chromosomal analysis, has transformed our understanding of leukemia biology^[9,10]^. These tools allow the identification of key genetic drivers of disease, such as the BCR-ABL1 fusion gene in ALL and FLT3 or NPM1 mutations in AML, which inform prognosis and guide therapeutic decisions^[11,12]^.

**HIGHLIGHTS**

- Genomic profiling enables early risk classification and guides personalized treatment strategies, improving survival and reducing relapse in pediatric leukemia.
- Minimal residual disease monitoring informs therapy intensity, allowing tailored interventions and minimizing toxicity.
- Targeted therapies, including tyrosine kinase inhibitor, monoclonal antibodies, and Chimeric Antigen Receptor T cells, enhance outcomes in high-risk or relapsed leukemia.
- Molecular diagnostics support stratified treatment plans, reducing overtreatment and preserving long-term health and quality of life.
- Integrated data from clinical trials and multi-omics approaches refine risk models and optimize individualized therapeutic decisions.

Minimal residual disease (MRD) monitoring has emerged as a cornerstone of modern risk assessment, enabling detection of leukemic cells at levels undetectable by conventional microscopy^[13,14]^. Integrating MRD status with molecular and clinical data allows for refined risk stratification, guiding the intensity of therapy and the use of targeted treatments. Furthermore, these approaches support the development of individualized treatment plans, reducing overtreatment in low-risk patients while intensifying therapy in those at higher risk of relapse^[15,16]^. The increasing understanding of leukemia biology has facilitated the development of targeted therapies, including tyrosine kinase inhibitors (TKIs) for Philadelphia chromosome-positive ALL, Chimeric Antigen Receptor T (CAR-T) cell immunotherapy for relapsed or refractory cases, and monoclonal antibodies such as blinatumomab and inotuzumab ozogamicin. Together, these advances provide opportunities for more precise, effective, and less toxic treatment, highlighting the ongoing shift toward personalized pediatric leukemia care^[17,18]^. This narrative review aims to provide an in-depth exploration of recent innovations in pediatric leukemia, focusing on advances in risk stratification, molecular profiling, targeted therapy, and the challenges associated with therapy resistance and long-term treatment complications.

## Aim

The aim of this review article is to provide an in-depth exploration of the recent advancements in the diagnosis, risk stratification, and targeted therapy for childhood leukemia.

## Methods

This narrative review was conducted through a structured and comprehensive search of peer-reviewed literature to explore recent innovations in pediatric leukemia care, with a focus on risk assessment, molecular profiling, targeted therapies, and associated challenges. Key electronic databases, including PubMed, Scopus, and Web of Science, were searched for articles published between 2015 and 2025 to capture contemporary advancements. Search terms included combinations of “pediatric leukemia,” “childhood leukemia,” “risk stratification,” “molecular profiling,” “targeted therapy,” “tyrosine kinase inhibitors,” “CAR-T cells,” and “precision medicine.”

The review prioritized studies reporting clinical outcomes, mechanistic insights, and translational research relevant to pediatric patients with ALL and AML. Both primary research articles, including clinical trials and cohort studies, and high-quality review articles were considered. Articles focusing on therapy resistance mechanisms, MRD monitoring, epigenetic therapies, immunotherapies, and long-term treatment complications were specifically included to provide a comprehensive understanding of current challenges and opportunities in personalized care.

Relevant studies were evaluated for their contribution to understanding molecular and genetic markers, risk-adapted therapy, treatment efficacy, and adverse effects. Emphasis was placed on integrating findings across preclinical, clinical, and translational studies to provide a holistic perspective of pediatric leukemia management. Information from included articles was synthesized narratively, highlighting trends, clinical implications, and emerging strategies, rather than performing a formal systematic review or meta-analysis. By using this narrative approach, the review aims to provide a detailed and clinically relevant summary of recent advances while identifying ongoing gaps and areas for future research in the field of pediatric leukemia care.

### Advancements in risk stratification

Risk stratification in pediatric leukemia has evolved significantly over the past several decades, moving from reliance on clinical parameters alone to a sophisticated integration of molecular, genetic, and MRD data. Historically, risk assessment incorporated factors such as age at diagnosis, initial WBC count, immunophenotype, and cytogenetic abnormalities, including t(12;21), t(9;22), and rearrangements of the MLL gene. While useful, these criteria were limited in predicting relapse and treatment failure, particularly among high-risk populations[19]. The incorporation of molecular and genomic profiling has transformed risk assessment by identifying genetic mutations and chromosomal abnormalities that directly influence prognosis. In ALL, the Philadelphia chromosome (BCR-ABL1) and IKZF1 deletions are associated with poor outcomes, whereas hyperdiploidy and ETV6-RUNX1 rearrangements often indicate favorable prognosis. In AML, mutations in FLT3, NPM1, and CEBPA serve as critical prognostic markers and inform therapy intensity. These molecular insights allow clinicians to classify patients more accurately and tailor treatment strategies based on individual disease biology^[20,21]^.

MRD monitoring has become a cornerstone of modern risk stratification. Defined as the small population of leukemia cells remaining after therapy, MRD is detectable using highly sensitive techniques such as flow cytometry, polymerase chain reaction (PCR), and NGS. Patients with detectable MRD following induction or consolidation therapy are at a significantly increased risk of relapse and often benefit from intensified treatment, including hematopoietic stem cell transplantation or targeted therapy. Conversely, low or undetectable MRD allows for treatment de-escalation, reducing toxicity without compromising outcomes. Quantitative MRD thresholds, such as 0.01% leukemic cells by flow cytometry, are increasingly integrated into risk-adapted protocols^[22,23]^. Beyond individual molecular markers and MRD, multi-omics approaches are emerging as a powerful tool to refine risk stratification further. Integrating genomic, transcriptomic, and proteomic data provides a more comprehensive understanding of leukemia biology, enabling the identification of novel molecular subtypes and therapeutic targets. For example, NGS studies in ALL have revealed subgroups with JAK-STAT pathway mutations or TP53 alterations, each associated with distinct clinical outcomes and therapeutic implications^[24,25]^.

Modern risk stratification models combine clinical, cytogenetic, molecular, and MRD data to classify patients into low-, intermediate-, or high-risk categories. High-risk patients – such as those with BCR-ABL1, high MRD levels, or FLT3-ITD mutations – are considered for intensified therapy, including targeted agents or stem cell transplantation, while low-risk patients receive fewer intensive regimens to minimize treatment-related toxicity. These precision-based approaches have led to improved survival, particularly among high-risk pediatric populations who previously had poor outcomes with conventional chemotherapy alone (Table 1)[26].

**Table 1:** Key genetic and molecular markers in pediatric leukemia and their clinical implications

Marker/mutation	Leukemia subtype	Prognostic significance	Targeted therapy/clinical relevance
BCR-ABL1 (Philadelphia chromosome)	ALL	Poor prognosis; associated with high relapse risk	Tyrosine kinase inhibitors (imatinib, dasatinib, ponatinib)
IKZF1 deletions	ALL	Poor prognosis, high-risk stratification	Intensified chemotherapy, consideration for stem cell transplant
ETV6-RUNX1	ALL	Favorable prognosis	Standard risk-adapted therapy
Hyperdiploidy (>50 chromosomes)	ALL	Favorable prognosis	Standard therapy; risk-adapted adjustments
MLL (KMT2A) rearrangements	ALL/AML	Poor prognosis, particularly in infants	Risk-adapted intensive therapy
FLT3-ITD	AML	Poor prognosis, high relapse risk	FLT3 inhibitors (midostaurin, gilteritinib)
NPM1 mutations	AML	Favorable prognosis if FLT3-ITD absent	Standard chemotherapy, risk-adapted therapy
TP53 mutations	ALL/AML	High-risk, therapy-resistant	Consider clinical trials or experimental therapies

AML- Acute Myeloid Leukemia, ALL- Acute Lymphoblastic Leukemia, FLT3-ITD- FMS-like tyrosine kinase 3-internal tandem duplication, BCR-ABL1- a fusion gene.

### Targeted therapies in childhood leukemia

Targeted therapies have transformed the landscape of pediatric leukemia treatment by focusing on specific molecular drivers of disease rather than indiscriminately targeting rapidly dividing cells[27]. One of the most well-established examples is the use of TKIs in Philadelphia chromosome-positive (Ph+) ALL^[28,29]^. Agents such as imatinib, dasatinib, and ponatinib selectively inhibit the BCR-ABL1 fusion protein, markedly improving remission rates and overall survival when combined with conventional chemotherapy[30]. However, resistance can emerge through point mutations in the BCR-ABL1 kinase domain, necessitating the use of next-generation TKIs or alternative therapeutic strategies. Continuous molecular monitoring is therefore critical to detect resistance early and guide treatment adjustments^[3,31]^. In AML, FLT3 mutations, particularly internal tandem duplications (ITDs), are associated with aggressive disease and poor prognosis. FLT3 inhibitors, such as midostaurin and gilteritinib, have been incorporated into treatment regimens to target this pathway. Similar to TKIs in ALL, resistance can develop through secondary mutations or pathway reactivation, highlighting the need for combination therapies and ongoing genomic surveillance.^[32–34]^

Immunotherapy has emerged as a powerful adjunct in pediatric leukemia care. CAR-T therapy, particularly targeting CD19 in relapsed or refractory ALL, has achieved remarkable remission rates in patients who have failed conventional therapy^[35,36]^. Monoclonal antibodies, including blinatumomab (anti-CD19) and inotuzumab ozogamicin (anti-CD22), have further expanded treatment options, demonstrating efficacy in both frontline and relapse settings. These therapies allow for selective targeting of leukemia cells, potentially reducing off-target toxicity associated with traditional chemotherapy.^[37–39]^ Epigenetic therapies represent an additional avenue for targeted intervention. DNA methyltransferase inhibitors, such as azacitidine and decitabine, and histone deacetylase (HDAC) inhibitors are being evaluated in pediatric leukemia to reverse aberrant epigenetic modifications that drive leukemogenesis and contribute to drug resistance. While still under clinical investigation, these agents may complement existing targeted therapies to overcome resistance mechanisms.^[4,40–42]^ Despite these advances, long-term complications remain an important consideration in pediatric leukemia care. Cumulative cardiotoxicity, particularly from anthracycline exposure, and the risk of secondary malignancies highlight the need for careful survivorship planning. Monitoring strategies and dose optimization are critical to minimize these adverse effects while maintaining treatment efficacy (Table 2)^[12,43,44]^.

**Table 2:** Overview of targeted therapies in pediatric leukemia

Therapy type	Mechanism/target	Leukemia subtype/indication	Clinical impact	Potential challenges/adverse effects
Tyrosine kinase inhibitors (TKIs)	Inhibit BCR-ABL1 fusion protein	Ph+ ALL	Improved remission and survival rates	Resistance due to kinase domain mutations
CAR-T cell therapy	Engineered T-cells targeting CD19	Relapsed/refractory ALL	High remission rates, potential curative option	Cytokine release syndrome, neurotoxicity, relapse from antigen escape
Monoclonal antibodies	Blinatumomab (anti-CD19), Inotuzumab ozogamicin (anti-CD22)	Relapsed/refractory ALL	Induces targeted cell death, reduces chemotherapy exposure	Infusion reactions, hematologic toxicity
FLT3 inhibitors	Target FLT3-ITD mutations	AML with FLT3 mutation	Improved event-free survival in combination therapy	Resistance via secondary mutations, limited long-term data
Epigenetic modulators	DNMT inhibitors (azacitidine, decitabine), HDAC inhibitors	ALL/AML in trials	Restore normal gene expression, may overcome resistance	Still experimental, variable efficacy, cytopenias

AML- Acute Myeloid Leukemia, ALL- Acute Lymphoblastic Leukemia, FLT3-ITD- FMS-like tyrosine kinase 3-internal tandem duplication, BCR-ABL1- a fusion gene.

### Therapy resistance and long-term complications in pediatric leukemia

Despite remarkable advances in targeted therapies and risk-adapted treatment strategies, therapy resistance remains a significant challenge in pediatric leukemia. In ALL, resistance to tyrosine TKIs can arise through mutations in the BCR-ABL1 fusion gene, which alter the kinase domain and reduce drug binding affinity.^[45–47]^ These mutations may emerge during treatment or at relapse, limiting the efficacy of first- and second-generation TKIs and necessitating alternative approaches, including the use of third-generation inhibitors or combination therapies. Similarly, in AML, mutations in the FLT3 gene – particularly ITDs – are associated with aggressive disease and can confer resistance to FLT3 inhibitors. The dynamic evolution of these mutations under selective therapeutic pressure highlights the need for continuous molecular monitoring and adaptive treatment strategies to preempt relapse^[48,49]^.

In addition to resistance, long-term complications of leukemia therapy are increasingly recognized as critical considerations in pediatric care. Conventional chemotherapy and targeted therapies can lead to cumulative cardiotoxicity, manifesting as long-term impairments in cardiac function, especially in patients exposed to anthracyclines or radiation. Secondary malignancies, including therapy-related myeloid neoplasms, may also arise years after initial treatment, representing a serious late effect of intensive therapy. These complications underscore the importance of integrating long-term survivorship monitoring into pediatric leukemia care, balancing the need for disease eradication with the preservation of overall health and quality of life.^[50–52]^

### Challenges and future directions

Despite remarkable progress in pediatric leukemia treatment, several challenges persist that limit the full potential of precision medicine. Therapy resistance remains a major obstacle, particularly in high-risk subtypes. In ALL, mutations in the BCR-ABL1 kinase domain can reduce the efficacy of TKIs; whereas in AML, FLT3 ITD mutations can confer resistance to FLT3-targeted therapies. The dynamic evolution of these mutations under therapeutic pressure underscores the need for continuous molecular monitoring and adaptive treatment strategies. Resistance mechanisms may also arise from leukemic stem cells (LSCs) that survive conventional therapies, contributing to relapse and highlighting the importance of targeted approaches against these reservoirs.^[45–47]^ Long-term complications of treatment represent another critical concern. Cumulative cardiotoxicity, particularly from anthracyclines, can impair cardiac function over time, and secondary malignancies may develop years after initial therapy. These late effects necessitate careful treatment planning, dose optimization, and lifelong survivorship monitoring to balance disease eradication with quality of life.^[53–55]^

Emerging strategies offer potential solutions to these challenges. Multi-omics integration – including genomic, transcriptomic, and proteomic profiling – can refine risk stratification, identify novel therapeutic targets, and enable early detection of resistance. Advances in immunotherapy, such as next-generation CAR-T cells and bispecific antibodies, are being developed to overcome resistance and achieve durable remissions. Epigenetic modulators and combination therapies targeting leukemic signaling pathways are under active investigation to prevent relapse and improve outcomes in resistant disease^[50,51]^. Furthermore, incorporating artificial intelligence and machine learning into clinical decision-making holds promise for personalizing treatment, predicting resistance, and optimizing therapy intensity. Expanding access to precision diagnostics and targeted therapies in low- and middle-income countries remains a key priority to ensure equitable improvements in pediatric leukemia outcomes^[55,56–58]^.

## Conclusion

Recent advances in pediatric leukemia care have transformed outcomes through the integration of molecular and genetic profiling, MRD-guided risk stratification, and targeted therapies. These innovations have enabled clinicians to tailor treatment intensity, improve survival rates, particularly among high-risk patients, and reduce chemotherapy-related toxicity. Targeted approaches – including TKIs for Philadelphia chromosome-positive ALL, CAR-T cell therapy for relapsed or refractory cases, and monoclonal antibodies such as blinatumomab and inotuzumab ozogamicin – have significantly improved remission rates and expanded therapeutic options. Despite these advances, important challenges persist, including the emergence of therapy resistance (e.g., BCR-ABL1 mutations in ALL and FLT3 mutations in AML) and long-term adverse effects, such as cardiotoxicity and secondary malignancies. Future strategies should focus on integrating multi-omics data, refining immunotherapeutic approaches, expanding access to precision diagnostics and therapies, and enhancing survivorship monitoring to further personalize care and optimize long-term outcomes.
